# Future Perspectives in Detecting *EGFR* and *ALK* Gene Alterations in Liquid Biopsies of Patients with NSCLC

**DOI:** 10.3390/ijms22083815

**Published:** 2021-04-07

**Authors:** Daniela Ferreira, Juliana Miranda, Paula Martins-Lopes, Filomena Adega, Raquel Chaves

**Affiliations:** 1 Biosystems and Integrative Sciences Institute (BioISI), Faculty of Sciences, University of Lisboa, 1749-016 Lisbon, Portugal; daniela_p_ferreira@hotmail.com (D.F.); julicmiranda26@gmail.com (J.M.); plopes@utad.pt (P.M.-L.); filadega@utad.pt (F.A.); 2 Department of Genetics and Biotechnology (DGB), University of Trás-os-Montes e Alto Douro (UTAD), 5000-801 Vila Real, Portugal

**Keywords:** non-small-cell lung cancer, *EGFR*, *ALK*, predictive biomarkers, liquid biopsies, next-generation sequencing

## Abstract

Non-small-cell lung cancer (NSCLC) is a major cause of death worldwide. Alterations in such genes as *EGFR* and *ALK* are considered important biomarkers in NSCLC due to the existence of targeted therapies with specific tyrosine kinase inhibitors (TKIs). However, specific resistance-related mutations can occur during TKI treatment, which often result in therapy inefficacy. Liquid biopsies arise as a reliable tool for the early detection of these types of alterations, allowing a non-invasive follow-up of the patients. Furthermore, they can be essential for cancer screening, initial diagnosis and to check surgery success. Despite the great advantages of liquid biopsies in NSCLC and the high input that next-generation sequencing (NGS) approaches can provide in this field, its use in oncology is still limited. With improvement of assay sensitivity and the establishment of clinical guidelines for liquid biopsy analysis, it is expected that they will be used in routine procedures. This review focuses on the usefulness of liquid biopsies of NSCLC patients as a means to detect alterations in *EGFR* and *ALK* genes and in disease management, highlighting the impact of NGS methods.

## 1. Introduction

Lung cancer is one of the major causes of death worldwide, counting more deaths than breast, prostate and colorectal cancers together [1]. The absence of symptoms in the initial disease stage is responsible for its underdiagnosis and consequently for the low survival rates observed [2]. Non-small-cell lung cancer (NSCLC) represents about 85% of all lung cancers [3], having a wide range of therapeutic options when compared to small-cell lung cancer (with a 15% incidence and poor prognosis) [4].

The mutation(s)/alteration(s) in specific genes, such as *EGFR* (epidermal growth factor receptor) or *ALK* (anaplastic lymphoma kinase) (among others), in NSCLC are considered predictive biomarkers since they can be used as targets for specific tyrosine kinase inhibitor (TKI) therapy, being also indicative of sensitivity or resistance to specific TKI treatments [5,6]. Although these biomarkers are scrutinized in the initial diagnosis (usually by PCR and Sanger sequencing methods) to define the therapy according to the molecular profile, a high number of patients acquire resistance during treatment. For example, the *EGFR* mutation p.T790M occurs in more than 50% of NSCLC patients treated with the first- and second-generation EGFR TKIs, resulting in therapy inefficacy [7,8,9].

The therapeutic choice in NSCLC is based on the phenotypic and molecular characterization of a single tumor biopsy (solid biopsy that is usually formalin-fixed and paraffin-embedded (FFPE)). Nevertheless, despite the high sensitivity of solid biopsies analysis, they are highly invasive and underestimate tumor heterogeneity and clonal evolution, that may ultimately result in therapeutic resistance, being also highly invasive [10]. Liquid biopsies emerged as a reliable approach to overcome these issues, still requiring further research considering sensitivity. They are defined as the use of patient’s body fluids (e.g., blood, urine, saliva and pleural effusion) to determine tumor-specific alterations [11]. Up until now, the analysis of tumor cell-free DNA (ctDNA) or circulating tumor cells (CTCs) present in the bloodstream were not used as routine practice in clinic, mostly due to the lack of validation methods. However, liquid biopsies are considered to be a promising molecular diagnosis tool and they have a further potential to be used in patient follow-up in order to evaluate therapy efficiency in a “real-time” mode [11,12,13]. Other putative applications have been indicated for liquid biopsies, including cancer screening, diagnosis or scrutiny of surgery success [1,2]. Additionally, new methods that emerged in the next-generation sequencing (NGS) era brought renewed hope, since they offer higher sensitivity power required to analyze these types of samples [3]. Although different PCR-based approaches have been approved for the analysis of *EGFR* mutations in liquid biopsies for many years, only recently, NGS panels were recognized by the FDA (Food and Drug Administration) for this purpose in NSCLC patients. Nevertheless, in order to apply this approach for routine clinical use, the validity of liquid biopsies’ analysis as being representative of the tumor itself needs to be further proved. Moreover, the scientific community needs to define evaluation criteria and to standardize the analytical methods [4,5].

Here, we present an update on the perspective of detecting *EGFR* and *ALK* gene alterations in liquid biopsies of patients with NSCLC, focusing on new methods that have emerged in the past few years and on the impact of NGS.

## 2. Alterations in *EGFR* and *ALK* Genes as Predictive Biomarkers in NSCLC

Currently, alterations in *EGFR* and *ALK* genes are considered important predictive biomarkers in NSCLC [5,6], since their identification in the tumor genome can determine a better outcome if a targeted therapy is implemented [14].

### 2.1. Mutations in EGFR as Predictive Biomarkers and EGFR TKIs in NSCLC

The *EGFR* gene is constituted by 28 exons and locates on the short arm of chromosome 7, encoding a 170-kDa transmembrane glycoprotein [6], with important functions in cell signal transduction [7]. The influence of EGFR in the pathogenesis of different cancer types has been widely studied, being used as a biomarker in the profiling of different tumor types [8]. Regarding NSCLC, mutations in *EGFR* are considered to be standard predictive biomarkers being indicative of treatment response and constituting the basis for the development of alternative targeted therapies, namely, EGFR tyrosine kinase inhibitors (EGFR TKIs) [9,10].

**Figure 1:**
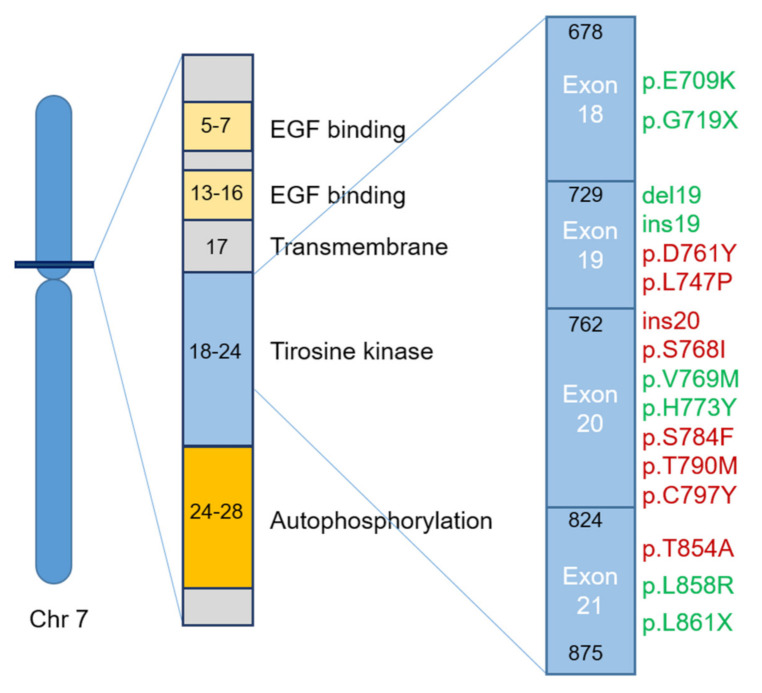
Effect of the most common *EGFR* mutations in NSCLC (Non-small-cell lung cancer) on the EGFR TKI (Tyrosine Kinase Inhibitor) response. Schematic representation of the location of the most frequent *EGFR* mutations in NSCLC and their relationship to resistance (red) and sensitivity (green) in the treatment with EGFR TKIs.

The most frequent mutations of the *EGFR* gene in NSCLC patients are located in four tyrosine kinase domain coding exons (in the ATP-binding region)—exons 18, 19, 20 and 21 (Figure 1), which are considered as activators since they result in an increase of the receptor kinase activity [11,12]. These mutations trigger the PI3K/AKT and JAK/STAT cellular pathways in tumor cells, resulting in cell proliferation, invasion and metastatic capacity, neovascularization and apoptosis evasion [11,13]. They can be classified in three different types, with classes I and II containing most of the mutations responsible for the sensitivity to EGFR TKIs [14]:

- Class I—in-frame deletions in exon 19. The most frequent mutations affect amino acids from L747 to E749 codons (Leu-Arg-Glu fragment, commonly known as LRE fragment)−p.delE746-A750 followed by p.delL747-P753insS, p.delL747-T751 and p.delL747-A750insP [15]. However, mutations can occur in all the amino acids encoded by exon 19, from E746 to D761. The different mutation types exhibit different therapeutic responses regarding the first-generation EGFR TKIs, being more effective in p.delE746 than in p.delL747 [15].
- Class II—single nucleotide substitutions can occur in exons 18, 20 and 21. The most frequent mutations in exon 18 of *EGFR* in NSCLC are p.E709X and p.G719X (X can be replaced by A, S, C and D) [16,17]. These mutations can be present in combination with other additional point mutations, such as p.S768I (exon 20) and p.L819Q (exon 21), reducing the sensitivity to the second-generation TKIs, specifically, afatinib and neratinib [18]. Frequently, in *EGFR* exon 20, a second mutation may occur—p.T790M, causing more than 50% of resistance acquisition to the first- and second-generation EGFR TKI drugs in NSCLC [19,20,21]. This last mutation increases the affinity to ATP and decreases the efficacy of ATP competitors, such as gefitinib and erlotinib [18,22]. In about 20–40% of NSCLCs, a third mutation, p.C797S, occurs in exon 20, resulting in resistance to the third-generation EGFR TKIs [23]. One of the most frequently observed *EGFR* mutation in NSCLC occurs on exon 21—p.L858R [24]. The p.L861Q mutation is also observed, but in a smaller number of cases, conferring sensitivity to EGFR TKIs [25,26].
- Class III—in-frame duplications and/or insertions in *EGFR* exon 20, 3 to 21 bp between amino acids 762 and 774 of EGFR [27]. The insertions in this exon (Ins20) are associated with TKI resistance and the worst prognosis in NSCLC patients [28].

EGFR-specific TKI drugs are small molecules and monoclonal antibodies used as anti-EGFR therapies. Small molecules reversibly compete with ATP (adenosine-5′-triphosphate), binding to the EGFR’s intracellular tyrosine kinase domain and, thus, inhibiting its autophosphorylation and downstream signaling pathways. Monoclonal antibodies inhibit EGFR activity, acting as a competitor for selective binding to the EGFR receptor’s inactive extracellular domain and block EGFR activation induced by the ligand [9,29]. To date, four generations of EGFR TKIs have been used or are being tested in clinical trials of NSCLC treatment [30].

The first-generation of EGFR TKIs (erlotinib and gefitinib) reduce autophosphorylation and receptor activation, resulting in tumor regression through apoptosis induction and inhibition of proliferation and angiogenesis [9]. The resistance observed in NSCLC patients treated with these EGFR TKIs resulted in the development of the second-generation of TKIs, such as afatinib, canertinib, dacomitinib, neratinib and pelitinib. These are irreversible inhibitors of EGFR which bind to a high-affinity receptor and irreversibly alkylate the Cys-773 residue (at the ATP-binding pocket). Afatinib is considered the first-line therapy for metastatic NSCLC, exhibiting the following *EGFR* mutations: p.G719X, p.S768I, p.L858R, p.L861Q and exon 19 deletions [31]. The third-generation of EGFR TKIs (e.g., osimertinib and rociletinib) has a mutated form as the target, with particular specificity to p.T790M, acting through its irreversible inhibition [29]. Rociletinib is a small molecule that inhibits the most common forms of mutated *EGFR*, including exon 19 deletions, p.L858R and p.T790M, but it is not effective on exon 20 insertion forms [32]. Osimertinib inhibits some mutated forms of *EGFR*, being more efficient against *EGFR* mutations p.L858R and p.T790M than against the wild type [33]. Although these drugs present high efficacy, the acquisition of some mutations, such as p.C797S, compromises their effectiveness. To overcome the lack of efficiency of the previous drugs, the fourth-generation of EGFR TKIs has been developed [30,34]. EAI001 is a new allosteric inhibitor of EGFR activity derived from thiazole amide that binds to the ATP-binding site resulting in an inactive conformation of the protein C-helix. EAI045 is the first EGFR TKI described as efficient in the NSCLC treatment in patients with p.T790M and p.C797S mutations. Its combined use with cetuximab (a monoclonal antibody that blocks the dimerization of EGFR) results in an anti-proliferative response [30,35]. Other fourth-generation TKIs are being developed to tackle p.T790M and p.C797S mutations [30].

### 2.2. Alterations in ALK as Predictive Biomarkers and ALK TKIs in NSCLC

The *ALK* gene is located on the short arm of chromosome 2 and is composed of 29 exons, which encodes for a 220-kDa transmembrane tyrosine kinase protein, also known as the ALK tyrosine kinase receptor or CD246 [36]. This protein belongs to the insulin receptor family and is usually present in the central nervous system and in lung tumor tissues [37]. Although *ALK* alterations are present in only 3–7% of NSCLC; with respect to chromosome rearrangements, this gene has the most important subclass of genetic alterations that leads to oncogenesis in NSCLC [37]. ALK signaling in cancer cells occurs by three main mechanisms: gene fusion, gene amplification and point mutations [38].

**Figure 2:**
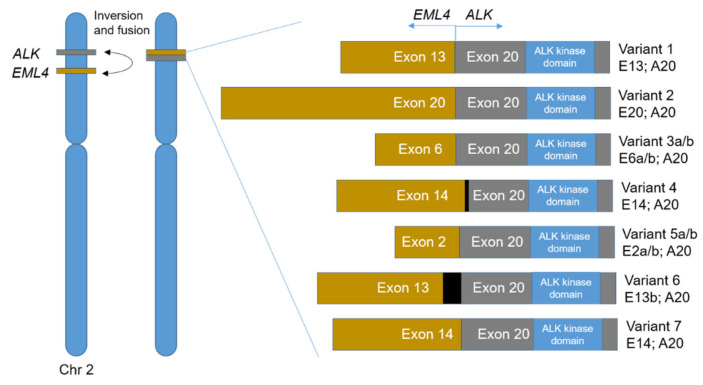
*EML4–ALK* fusion variants. Schematic representation of the *EML4–ALK* fusion event and the most common variants that can occur depending on the *EML4* gene breakpoint.

The fusion of *ALK* with other genes is the most common alteration, described in more than 30 gene partners, with the *EML4* (echinoderm microtubule-associated protein-like 4) gene being the most frequently found [39]. The first oncogenic fusion detected in lung cancer was *EML4–ALK*, resulting from a small inversion on the short arm of chromosome 2, that promotes the fusion of *EML4* N-terminal with *ALK* (exon 20) [40]. The EML4–ALK fusion protein presents several variants once the breakpoint in *EML4* can occur in different exons (2, 6, 13, 14, 15, 18 and 20). The most frequently found variants are 1 (E13; A20), 2 (E20; A20), 3 a/b (E6a/b; A20), 4 (E14; E20), 5 a/b (E2a/b; A20), 6 (E13b; A20) and 7 (E14; A20) (Figure 2) [37,40,41]. This fusion results in a constitutive activation of the ALK kinase domain, triggering intracellular signaling cascades related with cell proliferation and survival [42]. The expression of different EML4–ALK variants may influence the response to ALK TKIs, thus affecting the propensity for the development of secondary resistance-specific *ALK* mutations [43].

The point mutations detected in *ALK* of NSCLC patients represent about one third of the resistance mechanisms observed, being the most commonly found the p.L1196M (that is analogous to p.T790M of *EGFR*). Other mutations identified are p.1151Tins, p.L1152R, p.C1156Y, p.I1171T, p.F1174L, p.V1180L, p.G1202R, p.D1203N, p.S1206Y and p.G1269A [44]. These mutations increase the affinity of the tyrosine kinase receptor to ATP, decreasing TKIs’ binding affinity [45]. Mutations in the *ALK* gene, such as p.1151Tins and p.G1202R, result in resistance to the second-generation ALK TKIs; p.L1196M and p.L1152R confer sensitivity to the second- and third-generation ALK TKIs; and p.G1202R results in resistance to the first- and second-generation ALK TKIs [38].

The treatment of NSCLC patients with small-molecule ALK TKIs have presented excellent results regarding disease remission at the beginning of therapy. Three different generations of ALK TKIs were developed to date. Regarding the treatment with the first-generation ALK TKIs (crizotinib), resistance eventually occurs after 7–11 months of treatment, similarly to what occurs in EGFR TKI treatment [45,46,47,48]. In addition, 20–30% of ALK-positive patients develop metastasis during therapy (mainly at the central nervous system) [44]. To overcome this issue, the second- (ceritinib, alectinib, brigatinib and entrectinib) and third-generation (lorlatinib) ALK TKI drugs have been developed, with higher effectiveness and penetration capacity in the central nervous system [41]. Ceritinib and alectinib proved to be efficient against several crizotinib-resistant ALK-positive main NSCLC forms, including tumors with gatekeeper mutation p.L1196M [49,50]. However, ceritinib appears inefficient in tumors presenting the p.I1171T/N/S, p.F1174L/C, p.G1202R and p.G1269A mutations [38,45]. Brigatinib was developed as an effective and selective ALK inhibitor capable of overlapping the resistance mechanisms associated with crizotinib, presenting better results against almost all the *ALK* variants [44]. Lorlatinib is a third-generation ALK TKI also designed to overcome secondary mutations of *ALK* that confer resistance to treatment [51].

## 3. State of the Art on the Analysis of *EGFR* and *ALK* Mutations in Liquid Biopsies—Can Liquid Biopsies Be Used as a Routine Clinical Practice in NSCLC Patients in the Near Future?

The use of liquid biopsies in oncology has provided new approaches in the field of molecular diagnosis [3]. Different analytes are present in body fluids that result from cell secretion or cell component release as a result of tumor cells death. Particularly in the bloodstream, the following can be found and analyzed: cell-free DNA (cfDNA) or RNA (cfRNA), which can have a tumor origin and are known as circulating tumor DNA or RNA (ctDNA, ctRNA); circulating tumor cells (CTCs); and exosomes (EXOs) [3,52,53]. The analysis of these molecules enables point mutation detection, in-frame deletions and insertions, copy number alterations, translocations and epigenetic modifications frequently found in cancer. The detection of these last events is only possible because of the technological advances achieved in this research area, enhancing sensitivity of the assays used to detect cancer-specific mutations in these analytes. However, as different sources of tumor samples can be found in liquid biopsies, it is important to choose the best analyte for a particular purpose. The scrutiny of CTCs is an excellent approach to find cancer-specific alterations and to perform cell morphology analysis in advanced cancer; however, it is not suitable in early stages of the disease [1,3]. Exosomes and CTCs can also be a source of DNA and RNA, allowing the detection of mutations [54]. CtDNA is easy to obtain and presents several clinical applications since it gives an updated snapshot of the tumor due to its short half-life in circulation (between 16 min and 2.5 h), reflecting its heterogeneity and its evolution across time, even in tumors with difficult biopsy location [3,54,55]. Nevertheless, the amount of ctDNA present in the plasma can vary widely among patients [56] depending on different factors, such as, among other things, body weight, sport practice, tumor volume/stage, therapy [57,58]. CtDNA isolation should be performed using plasma instead of blood serum since it can be contaminated with the blood cells’ DNA [54].

As referred, the use of liquid biopsies is a very promising tool for molecular cancer diagnosis in patients, both in early and advanced stages of the disease. So far, four different clinical scenarios are anticipated for the potential use of liquid biopsies:

- Initial diagnosis—the analysis of some biomarkers in liquid biopsies allows the identification of mutations in targeted genes (e.g., *EGFR* and *ALK*), indicating the best therapy protocol [1,2];
- Checking surgery success—the scrutiny of predictive biomarkers in liquid biopsies can be indicative of therapy/surgery success. Due to the short lifetime of cfDNA, its presence indicates an incomplete surgical resection of the tumor or the presence of undetected tumor metastasis [1,2];
- Therapy monitoring—the use of liquid biopsies can be essential in the detection of early cancer recurrence (before radiographic or clinical detection), allowing an early treatment change. The detection of new mutations (not present in the primary tumor) is also possible, guiding the second-line therapy choice. Due to the non-invasive character of these biopsies, the patient follow-up along the treatment allows the detection of new mutations that can lead to resistance (e.g., *EGFR* p.T790M) [1,2]. Also, ctDNA presence during treatment revealed a significant increase in the progression-free survival from 55 to 295 days (non-ctDNA vs. ctDNA) [59];
- Cancer screening—the use of liquid biopsies has a potential to detect tumor presence before it can be clinically identifiable, reducing cancer morbidity and mortality. However, some problems related with its use in early cancer detection, such as overdiagnosis and high rate of false positives, have to be overcome [1,2].

Thus, the use of liquid biopsies in cancer screening, diagnosis and monitoring is undoubtedly advantageous in the future oncology practice and, for now, it should be used as a complementary analysis of solid biopsies (Table 1) [1,2,60].

The *EGFR* mutation analysis in NSCLC patients’ liquid biopsies (namely, ctDNA) revealed to be highly correlated with treatment response when using the third-generation EGFR TKIs in several clinical trials [5,61,62,63]. Furthermore, *EGFR* mutation p.T790M was detected in ctDNA samples before the first signs of resistance to treatment occurred, allowing an early change of therapy [61]. In a study by Taus et al., the *EGFR* mutation profile change using plasma samples allowed to predict response in 93% and progression in 89% NSCLC patients before radiological assessment [64].

Although blood is the gold standard of liquid biopsies, saliva, urine or pleural fluids can also be used for predictive biomarker detection in NSCLC. Urine and saliva are particularly easy to collect, with no requirement of technical/medical procedures and independent on the patients’ status [65]. However, and differently to what occurs with cfDNA in blood that is protected by nucleoprotein complexes or extracellular vesicles, the cfDNA present in urine and saliva can be cleaved by nucleases, originating shorter fragments [66,67]. This fact, together with the low abundance of ctDNA (<0.01%) in these two biological samples, are major challenges that need to be considered before applying them in NSCLC mutation analysis [65]. Nevertheless, the genomic profile of cfDNA extracted from plasma, urine and saliva was highly correlated to that of tissue samples [68]. Many studies on *EGFR* mutation detection in urine [69,70,71], saliva [67,72,73] and pleural fluids [74,75,76] showed promising results. Detection sensitivity of *EGFR* mutations p.T790M, p.L858R and del19 in plasma and urine samples was compared to that in tumor tissue, revealing 93% and 72% sensitivity, respectively. Yet, the highest accuracy was obtained with a combined analysis [70]. Another study regarding *EGFR* mutation detection in plasma, saliva and urine demonstrated that sensitivity can be improved by the combination of these three fluids (from 84% in plasma to 91% in the combined approach) [72]. However, saliva seems to be the fluid providing the lowest sensitivity in *EGFR* mutation detection, as referred by Wu et al. [72] and another study [73]. Regarding the analysis of cfDNA in pleural fluids, a good correlation was found between the mutation profiles present in plasma [77] and tumor [78] samples. However, more research in this field is required to implement these methodologies in clinical procedures.

Liquid biopsies can potentially be used as non-invasive and reliable clinical tools in the identification of predictive biomarkers to trace the molecular profile of cancer patients in real-time, allowing to adapt treatment plans to each disease stage. This can be used to administer personalized and targeted therapies [53]. Still, the major limitations in the ctDNA routine use are the sensitivity of the mutation detection and the lack of consensus considering evaluation criteria and method standardization [4,5]. Liquid biopsies can only become a reality in cancer patients’ healthcare if the scientific community joins forces to develop and validate tools which will allow the use of a sample that mimics the tumor itself, considering the heterogeneity of the tumor, with high sensitivity. In fact, different methods have been established to analyze predictive biomarkers in liquid biopsies. Moreover, the emergence of NGS-based technologies has increased the interest in liquid biopsies despite their sporadic use and the remaining method validation required for routine use in cancer patients [3].

### EGFR and ALK Alteration Detection in Liquid Biopsies—The Input of NGS in ctDNA Analysis

Different methods have been developed to detect mutations in *EGFR* and *ALK* genes in NSCLC patients’ liquid biopsies during initial therapy definition and in the patient’s follow up response during TKI treatments, anticipating mutation detection associated with therapy resistance at an early stage [79].

Highly specific and sensitive techniques commonly used in FFPE samples, such as immunohistochemistry or fluorescence in situ hybridization, cannot be used in liquid biopsy analysis [80,81]. However, the use of PCR-based methods (polymerase chain reactions, namely, real-time PCR and digital PCR) and NGS (next-generation sequencing) appeared to be of great value to scan for mutations in ctDNA [5,81,82,83]. The method of choice depends not only on the analyte (and its quantity), but also on the main objective, that is, whether it is used for diagnosis, detection of specific mutations, monitoring therapy response or surgical success assessment [55]. Several assays have been established (some of them commercially available) to detect alterations in *EGFR* and *ALK* genes from liquid biopsies of NSCLC patients. The sensitivity of the method used to identify ctDNA mutations is a key point in liquid biopsy accuracy since the ctDNA level in a sample is about 1% and the mutated alleles are supposed to occur with a frequency below 0.01%, which may not be detected due to the method’s limitations. Thus, classical sequencing methods (Sanger sequencing) are not adequate to detect allele variants due to their low sensitivity (>10%) [54]. Some PCR-based methods, such as digital PCR (dPCR) and droplet dPCR (such as BEAMing—beads, emulsion, amplification and magnetics), are useful for common mutation detection and quantification with high sensitivity (<0.001%), varying with ctDNA quantity [54,84]. However, NGS-based approaches allow the analysis of (1) a panel containing specific gene mutations with very high sequencing depth (high specificity and sensitivity) known as targeted sequencing, (2) whole-exon (exome) sequencing and (3) whole genome sequencing [5]. The first method developed for targeted sequencing was named TAm-Seq (for tagged amplicon sequencing), described by Forshew et al., consisting in a two-step amplification process (that generates tagged amplicons) followed by a subsequent analysis by NGS. With this, a gene panel is analyzed with a sensitivity and specificity higher than 97% [85]. Furthermore, it allows the identification of predefined mutations, such as the ones related with cancer therapy and outcome, at an allele frequency (AF) of 0.14%. The eTAm-Seq (enhanced TAm-Seq) is an improved version allowing the identification of hotspot mutations (such as in *EGFR* and *ALK*), covering 35 genes with 90% detection sensitivity, in mutated alleles with a frequency of 0.25% [54]. Other targeted NGS-based methods/assays were developed focusing on the increase of detection specificity and sensitivity. Whole genome or exome sequencing methods are less sensitive; however, they are recommended for de novo mutation detection, chromosomal aberrations and clonal evolution studies [54]. Nevertheless, as some mutations are single-base changes and as ctDNA amount is limited, deep sequencing of gene panels seems to be more adequate for liquid biopsy analysis [55]. Even so, the relative inaccuracy of NGS can result in errors that can be misinterpreted as mutations, but that can be overcome with specific strategies (i.e., two-strand sequencing, use of molecular identifiers or labeling individual input DNA molecules) that increase sensitivity, allowing to detect cfDNA mutated alleles with a frequency lower than 0.1% [55,86]. In addition to the implementation of good laboratory practices, ctDNA extraction protocols (initial amount), the depth sequencing average and the algorithms underlying the software used to detect mutations are critical aspects for the establishment of a reliable, sensitive and standard method [79]. The use of liquid biopsies in NSCLC is already approved for clinical practice; however, their employment is still limited in comparison to the potential. The application of the most suitable targeted therapy in respect to a specific *EGFR* mutation has demonstrated to be essential in order to obtain the best disease outcome. Thus, treatment design needs to be based on tumor profiling, and in the cases where solid tumor biopsy is difficult to obtain, liquid biopsies with ctDNA analysis can be crucial, allowing at the same time treatment follow-up and detection of new resistance-related mutations at an early therapy stage [3].

Although NGS-based approaches are being implemented in the liquid biopsy analysis and have been recently recognized by the FDA, PCR-based methods used to detect *EGFR* mutations in ctDNA samples from NSCLC patients were previously approved and validated by the FDA and the EMA (European Medicines Agency) [54,87]. In 2015, the EMA approved the use of Therascreen EGFR RGQ PCR Kit (QIAGEN, Hilden, Germany), an ARMS-based (Amplification refractory mutation system) assay used for ctDNA-based diagnosis in patients where tumor biopsy is difficult or even unattainable [61,88]. This assay detects up to 42 mutations in exons 18, 19, 20 and 21 of *EGFR* (including deletions in exon 19, p.T790M, p.L858R and p.L861Q) in both tissue and liquid biopsy samples [89]. Later, in 2016, the FDA approved the use of Cobas EGFR Mutation Test v2 (Roche Molecular Systems, Inc., Pleasanton, CA, USA) applied to liquid biopsies to detect *EGFR* mutations in ctDNA samples for the same purpose [88,90]. However, none of these assays are NGS-based. Other assays that have been developed to screen *EGFR* mutations (PANAMutyper R EGFR, Droplet digital PCR, OncoBEAM) are also PCR-based techniques [5,89]. Nonetheless, NGS-based panels can offer an unique opportunity to increase the number of analyzed mutations in a unique assay, with higher throughput and sample efficiency use (small amount) than those obtained using PCR-based methods (Table 2) [5].

Several clinical trials have been conducted to prove clinical validity of ctDNA analysis considering *EGFR* mutation screening in NSCLC patients. The IGNITE and ASSESS trials tested the efficacy of *EGFR* mutation detection in plasma from NSCLC patients and reported limited sensitivity (compared to mutations found in tissue samples) (<50%) [91,92]. However, it is important to notice that these sensitivity data widely vary between different countries in these trials (36–100%), highlighting the urgency in developing standard methods for ctDNA analysis [54]. In a subgroup analysis regarding Therascreen EGFR RGQ PCR Kit and Cobas EGFR Mutation Test for *EGFR* screening, a high concordance between plasma and tissue was observed (95% and 96%), with a sensitivity rate of 73% and 75%, respectively, and 99% and 100% of specificity [93]. The IFUM clinical trial tested the efficacy of gefitinib in NSCLC patients and the validity of Therascreen EGFR RGQ PCR Kit for ctDNA analysis. The concordance with tissue *EGFR* mutation was 94.3% and the sensitivity and specificity were 65.7% and 99.8%, respectively [94]. The AURA trials demonstrated the clinical potential of ctDNA testing for *EGFR* mutation p.T790M detection in plasma from NSCLC when the efficacy, dose and safety of osimertinib were evaluated. This trial reported the p.T790M detection sensitivity/specificity in plasma for Cobas *EGFR* Mutation Test (93%/100%), OncoBEAM (81%/69%), ddPCR (71%/83%) and Therascreen EGFR RGQ PCR Kit (29%/100%) [61,62,63,95,96]. Plasma and tissue samples showed to be concordant for up to 74% in the detection of p.T790M [61,62,96]. The low sensitivity observed in these trials raised some questions about the reliability of liquid biopsies for routine use in clinical practice that require further standardization, optimization and validation [54].

Considering *ALK* gene alteration analysis in NSCLC patients’ plasma, some assays have been designed. Nevertheless, the mutation detection sensitivity in these cases is lower than in the assays used for *EGFR*. New NGS-based approaches are being developed for the *ALK* gene, which facilitate *ALK* gene fusion detection using ctDNA or ctRNA (not highly fragmented) samples demonstrating high sequencing coverage and sensitivity for this particular situation [97,98,99].

Regarding targeted NGS panels, two assays were approved in 2020 by the FDA suitable to screen several mutations in NSCLC-related genes, including *EGFR* and *ALK*, in liquid biopsies:

- Foundation-One Liquid CDx (Foundation Medicine, Cambridge, MA, USA)—designed to detect alterations, such as point mutations, insertions and deletions, in 311 genes, rearrangements in four genes, and copy number variations in three genes using a targeted high-throughput hybridization-based capture technology [5];
- Guardant360 CDx (Guardant Health, Redwood City, CA, USA)—hybrid capture-based deep sequencing of defined regions in 74 genes reducing the number of false positives through the use of individually tagged cfDNA libraries [100].

The implementation of *EGFR* and *ALK* alteration analysis using ctDNA samples requires a detailed consideration of several aspects, such as, among other things, the ctDNA extraction method, plasma amount required to extract ctDNA, ctDNA quantity required to perform the assay, plasma conservation tubes used (EDTA vs. cfDNA preservation tubes) and sample processing time or plasma storage methods.

Proficiency studies considering *EGFR* mutations were performed at different laboratories, showing that mutation detection is quite promising; however, some discrepancies found between the laboratories have conditioned the implementation of liquid biopsies in cancer analysis [5,54]. Thus, the systemic use of liquid biopsies in clinical practice requires overcoming several obstacles, such as lack of standardization (use of different high-throughput analytical platforms), lack of sensitivity and specificity (associated with low ctDNA recovery) and high cost (including infrastructure and human resources). The relevance of liquid biopsies has been increasing in association with the advances in NGS techniques, which have generated large datasets obtained using liquid biopsies, helping to validate blood-based tumor biomarkers. Although some technical issues still need to be addressed, the designed assays are suitable for use in cancer evaluation since they are capable of detecting different types of mutations in cancer biomarker genes, such as point mutations, insertions or deletions (e.g., in *EGFR*, *ALK*, among others), translocations/gene fusions (e.g., *EML4–ALK*), copy number variations and epigenetic alterations [3].

## 4. Conclusions

Emergence of NGS-based approaches has boosted the potential use of liquid biopsies for mutation detection in several genes used as cancer biomarkers, such as *EGFR* and *ALK*, allowing the establishment of guidelines in targeted therapies using TKIs in NSCLC. The perspective of ctDNA use in prognostic, diagnostic and predictive testing using NSCLC-associated biomarkers is expected to become a reality in routine clinical procedures in the near future. These strategies will provide NSCLC patients a highly sensitive minimal invasive methodology capable of translating tumor heterogeneity.

## Figures and Tables

**Table 1:** Characteristics of liquid and solid biopsies (FFPE tumors) [11,12,67,68].

	Liquid Biopsies	Solid Biopsies
**Invasiveness**	No	Yes
**Continuous molecular assessment of the tumor**	Biological evaluation of the tumor at any time allowing therapy monitoring	Difficulties in the follow-up of the tumor evolution
**Cost**	Low	High
**Biopsy collection**	Easy; simple blood (or urine, saliva) collection	Difficult; small tumors may require multiple attempts to retrieve enough tissue
**Tumor heterogeneity**	Allow examining the longitudinal evolution of the tumor; better reflect tumor heterogeneity	Only allow for a snapshot in time of the ever-evolving tumor biology; limited access to the intra- and intertumor heterogeneity
**Specificity**	High; overdiagnosis in early cancer detection and high rate of false positives are observed	Higher; they allow the application of several specific methods

**Table 2:** Summary of the PCR-based and NGS-based methods’ characteristics [55].

Method	Advantage	Disadvantage	Clinical Application	Validated Assays for NSCLC Liquid Biopsies
PCR-based	Sensitivity (AF), 0.1–0.001%; straightforward data analysis; rapid; lower cost.	Need prior knowledge of the mutation; analysis limited to few targets; higher amount of sample.	Analysis of specific gene mutations (as in *EGFR*) in NSCLC patients’ liquid biopsies	Therascreen EGFR RGQ PCR Kit and Cobas EGFR Mutation Test v2
NGS-based	No need in prior knowledge of the mutation; analysis of a high number of targets; smaller amount of sample.	Sensitivity (AF), 5–0.1%; needs extensive bioinformatics support for data analysis; slower; high cost.	Analysis of several gene mutations/alterations (as in *EGFR* and *ALK*) in NSCLC patients’ liquid biopsies	Foundation-One Liquid CDx and Guardant360 CDx

## Data Availability

Not applicable.
